# Mitochondrial dysfunction and oxidative stress in heart disease

**DOI:** 10.1038/s12276-019-0355-7

**Published:** 2019-12-19

**Authors:** Jessica N. Peoples, Anita Saraf, Nasab Ghazal, Tyler T. Pham, Jennifer Q. Kwong

**Affiliations:** 1 Department of Pediatrics, Division of Cardiovascular Biology, Emory University School of Medicine, Atlanta, GA 30322 USA; 2 Department of Medicine, Division of Cardiology, Emory University School of Medicine, Atlanta, GA 30322 USA; 3 Emory College of Arts and Sciences, Emory University, Atlanta, GA 30322 USA

**Keywords:** Cardiovascular diseases, Cardiomyopathies

## Abstract

Beyond their role as a cellular powerhouse, mitochondria are emerging as integral players in molecular signaling and cell fate determination through reactive oxygen species (ROS). While ROS production has historically been portrayed as an unregulated process driving oxidative stress and disease pathology, contemporary studies reveal that ROS also facilitate normal physiology. Mitochondria are especially abundant in cardiac tissue; hence, mitochondrial dysregulation and ROS production are thought to contribute significantly to cardiac pathology. Moreover, there is growing appreciation that medical therapies designed to mediate mitochondrial ROS production can be important strategies to ameliorate cardiac disease. In this review, we highlight evidence from animal models that illustrates the strong connections between mitochondrial ROS and cardiac disease, discuss advancements in the development of mitochondria-targeted antioxidant therapies, and identify challenges faced in bringing such therapies into the clinic.

## Heart disease: Signaling a halt to disease progression

Heart disease progression could be tackled by targeting signaling molecules that cause oxidative stress. Jennifer Kwong at Emory University School of Medicine in Atlanta, USA, and co-workers reviewed research into the role of mitochondria and their associated signaling molecules in the development of heart disease. Mitochondria are a major source of reactive oxygen species (ROS), signaling molecules involved in muscle contraction and calcium transfer in the heart, but they also destroy ROS to maintain a balance. Disruption to this balance can lead to elevated ROS, causing DNA and cellular damage, triggering disease. Animal trials using drugs to target mitochondrial ROS show promise in limiting heart disease progression. Further research is needed to determine whether this approach will work in humans and which specific heart problems might benefit from such therapies.

## Introduction

Reactive oxygen species (ROS), including the superoxide anion, the hydroxyl radical, and hydrogen peroxide, are critical signaling molecules with important roles in both cardiac physiology and disease. Both cytosolic sources, including NADPH oxidases (NOX), xanthine oxidase, cyclooxygenases, and cytochrome P450 enzymes, and mitochondrial sources, including the respiratory chain, monoamine oxidases (MAOs), p66shc, and NOX4, contribute to the intracellular ROS pool. Under physiological conditions, cardiac ROS signaling regulates heart development and cardiomyocyte maturation, cardiac calcium handling, excitation contraction coupling, and vascular tone (reviewed in greater detail in ref. ^1^). However, pathological conditions of unregulated ROS production leading to elevated ROS levels can result in oxidative stress through oxidative damage to DNA, proteins, and lipids, as well as activation of the mitochondrial-permeability transition pore (MPTP), mitochondrial dysfunction, and cell death^2^. Indeed, dysregulated ROS production and oxidative stress have been implicated in a host of cardiac diseases, including cardiac hypertrophy, heart failure (HF), cardiac ischemia–reperfusion injury (IRI), and diabetic cardiomyopathy (discussed in greater detail in refs ^3–6^).

Given the important roles of ROS signaling in both cardiac physiology and disease, ROS signaling is tightly regulated, and intracellular redox homeostasis must be maintained to ensure that physiological ROS signaling can occur while pathological ROS signaling pathways are not activated. Intracellular ROS levels are held in check by an intricate array of antioxidant defense systems that include superoxide dismutases, catalase, the glutathione peroxidase/reductase (GSH-PX) system, and the peroxiredoxin/thioredoxin (PRX/Trx) system. Similar to conditions of unregulated ROS production, an impairment in cellular antioxidant defenses and ROS scavenging can also lead to cardiac dysfunction^7–11^.

In light of the strong connections between ROS and cardiac disease, there has been intense interest in deciphering the mechanisms regulating cardiac ROS production and detoxification, as well as in developing therapies to limit ROS production and enhance ROS detoxification as a means to ameliorate cardiac disease outcomes. Importantly, there has been increasing evidence that strategies targeting global ROS activity, such as trials with systemic administration of the antioxidants vitamin C and vitamin E, have fallen short in their ability to mitigate cardiac disease^12^, while specific targeting of the mitochondrial ROS pool may be highly beneficial^13^. In support of a central role for mitochondria-derived ROS in cardiac disease pathogenesis, there has been strong support from in vivo models that link dysregulation of mitochondrial ROS production and/or impairment in mitochondrial ROS scavenging to cardiac dysfunction.

In this review, we discuss intracellular sources of ROS, the role of oxidative stress in heart disease, evidence linking mitochondrial ROS to cardiac disease, the efficacy of ROS-based therapeutics in the heart, and the emerging frontier of mitochondria-targeted antioxidant therapies and their application to cardiac disease.

## Mitochondrial sources of ROS

**Fig. 1:**
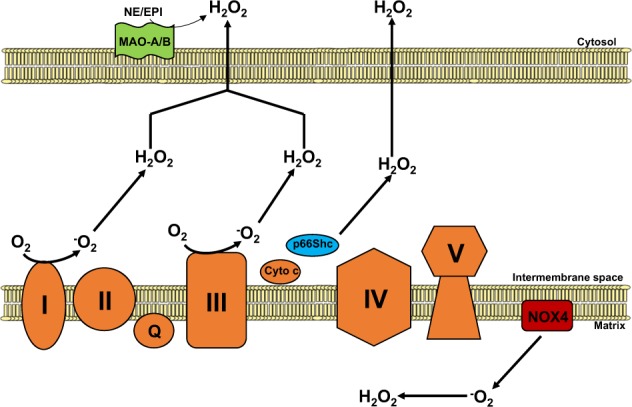
Mitochondrial ROS generation. Respiratory chain complexes I and III (orange) generate superoxide (O_2_^−^) and hydrogen peroxide (H_2_O_2_) from molecular oxygen (O_2_) within the mitochondrial intermembrane space. p66Shc (blue), in association with cytochrome *c*, participates in ROS signaling by producing hydrogen peroxide also within the intermembrane space. NADPH oxidase 4 (NOX4; red) localizes to the inner mitochondrial membrane, generating O_2_^−^ and H_2_O_2_ within the mitochondrial matrix. Monoamine oxidase isoforms A and B (MAO-A/B; green) degrade monoamines to aldehydes and H_2_O_2_ in the outer mitochondrial membrane.

The mitochondrial respiratory chain is central to energy production as it couples electron transfer between respiratory chain complexes to proton transport across the mitochondrial inner membrane to generate the electrochemical gradient required for ATP synthesis. In addition to being essential for energy production, the respiratory chain is also the predominant source of intracellular ROS, which are produced as a byproduct of electron transfer (Fig. 1). Although the precise sites of ROS production within each complex and the mechanisms by which they are produced are not fully elucidated, complexes I (NADH:ubiquinone oxidoreductase) and III (ubiquinol:cytochrome *c* oxidoreductase) are recognized as the major sources of ROS within the respiratory chain. For complex I, superoxide production has been suggested to stem from either reduced flavin mononucleotide^14^ or the N-1a and N-1b iron–sulfur clusters^15^. For complex III, superoxide has been suggested to be generated at the ubiquinol oxidation site^16,17^.

In addition to the respiratory chain, a number of additional mitochondria-localized proteins have been shown to contribute to the mitochondrial ROS pool. These proteins include p66shc,MAOs, and NOX4 (Fig. 1). P66shc is a member of the Shc (Src homology 2 domain and collagen-homology region) family of cytosolic adaptor proteins. Unlike its molecular relatives p52Shc and p46Shc, which regulate Ras signaling, p66shc has been found to play an important role in oxidative stress signaling. As a cytosolic protein that partially localizes to the mitochondrial intermembrane space^18^, p66shc contributes to mitochondrial ROS production by oxidizing cytochrome *c* and stimulating hydrogen peroxide production^19^.

The MAO isoforms A and B (MAO-A and MAO-B) have also been identified as important sources of mitochondrial hydrogen peroxide. Localized to the mitochondrial outer membrane, MAOs utilize the cofactor FAD to catalyze the oxidative degradation of monoamines, such as epinephrine and norepinephrine, into hydrogen peroxide and aldehydes. Importantly, both MAO-A and MAO-B are expressed in the heart and, as described in greater detail below, have been shown to play an important role in HF and cardiac IRI.

NOXs are a family of proteins involved in intracellular ROS production and catalyze the transfer of NADPH to molecular oxygen to generate superoxide and hydrogen peroxide. Of particular interest is NOX4, which partially localizes to the mitochondrial inner membrane in cardiomyocytes and renal cells^20–22^. NOX4 has been shown to be a source of mitochondrial superoxide and hydrogen peroxide as NOX4 knockdown reduces the production of both ROS species^22,23^. More recently, NOX4 activity has been found to be regulated by ATP, suggesting that NOX4 can couple mitochondrial oxidative stress signaling to a cellular energetic state^22^.

## Mitochondrial ROS scavenging systems

**Fig. 2:**
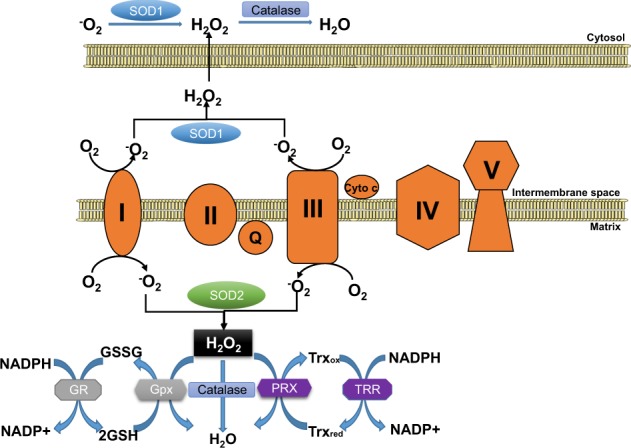
ROS scavenging systems in the mitochondria. Superoxide (O_2_^−^) is produced at respiratory chain complexes I and III and is dismutated by SOD1 (intermembrane space and cytosol) and SOD2 (matrix) to generate hydrogen peroxide (H_2_O_2_). Catalase, localized to both the cytosol and the mitochondrial matrix, converts H_2_O_2_ into H_2_O. Mitochondrial H_2_O_2_ detoxification can also be catalyzed by mitochondria-localized glutathione peroxidases (Gpx1 and Gpx4) and peroxiredoxins (PRX3 and PRX5). Gpxs oxidize glutathione (GSH) into GSSG, and reduced glutathione is regenerated by glutathione reductase (GR) using the reducing equivalents of NADPH. PRXs oxidize thioredoxin (Trx, with Trx2 being mitochondria-localized), and the reduced Trx pool is regenerated by the NADPH-dependent action of thioredoxin reductase (TRR; with TRR2 localized to the mitochondrial matrix).

To regulate oxidative stress created by mitochondrial ROS, mitochondria employ an intricate network of ROS scavenging systems that coordinately work to mitigate this stress (Fig. 2). These systems include superoxide dismutases (SODs), which convert the highly reactive superoxide radical into hydrogen peroxide, which is then further detoxified by catalase, the GSH-PX and the PRX/Trx system.

The first line of defense against mitochondrial ROS is the SODs, which dismutate superoxide into hydrogen peroxide_._ There are three different SOD isoforms (SOD1, SOD2, and SOD3) that display distinct cellular localizations, thereby controlling the compartment-specific ROS pools. SOD1, also known as copper-zinc SOD (Cu/Zn SOD), is mainly cytosolic but has also been found to localize to the mitochondrial intermembrane space. SOD2 (known as manganese SOD, MnSOD) localizes to the mitochondrial matrix, while SOD3 is found in the extracellular matrix. The control of the mitochondrial localization and activities of SOD1 and SOD2 are critical to mitochondrial ROS scavenging. For SOD1, it has emerged that intermembrane space targeting and activation is dependent on CCS1 (the copper chaperone of SOD1), which aids in enzyme maturation and activation^24,25^. In the case of SOD2, it has recently been shown that activity can be regulated by posttranslational acetylation, where acetylation can decrease activity while Sirt3-mediated deacetylation promotes ROS scavenging function^26^.

As mentioned above, catalase, the GSH-PX system and PRX/Trx are required for the breakdown of hydrogen peroxide. Catalase is an important peroxisomal antioxidant enzyme that catalyzes the degradation of hydrogen peroxide into water and oxygen. As peroxisomes are home to a number of ROS generating oxidases, peroxisomal catalase is a key component of the intracellular ROS detoxification system. In addition to the peroxisome, catalase has also been detected in heart mitochondria^27^, suggesting that it can also play a role in controlling the mitochondrial ROS pool.

GSH-PXs 1 and 4 (Gpx1 and Gpx4, respectively) are also localized to mitochondria and utilize reduced glutathione (GSH) to convert hydrogen peroxide into water, thus oxidizing GSH into glutathione disulfide (GSSG). GSSG is then reduced back into GSH by the activity of glutathione reductase, which requires NADPH to regenerate the mitochondrial pool of GSH (reviewed in greater detail in Andreyev et al.^28^). In addition to the enzymatic action of GPX, GSH itself is an important nonenzymatic antioxidant due to its ability to directly neutralize the hydroxyl radical and to regenerate the active forms of the antioxidant vitamins E and C^29^.

PRXs comprise a large and conserved family of peroxidases that have the ability to scavenge both hydrogen peroxide and peroxynitrite (reviewed in greater detail in Perkins et al.^30^). Within this family, PRX3 and PRX5 are found in the mitochondria, with PRX3 exhibiting mitochondrial matrix localization and PRX5 localizing to mitochondria, peroxisomes, and the cytosol. PRXs are oxidized during the detoxification of hydrogen peroxide into water and are converted into reduced PRXs by Trxs. Oxidized Trx is in turn reduced by the enzyme Trx reductase using NADPH as a cofactor. Of note, Trx2 is localized to the mitochondria and has been found to play an important role in limiting mitochondrial ROS production and regulating cardiac function^10,11^.

## Oxidative stress and heart disease

### IR injury

Ischemic cardiac injury resulting from the sudden occlusion of a coronary vessel as occurs in myocardial infarction induces a cascade of tissue hypoxia and cellular ATP depletion. Limiting the duration of hypoxia by restoring blood supply through percutaneous coronary interventions is now central to medical practice and has been shown to benefit survival. When myocardial ischemia exceeds 20 min, a transmural “wave front” of myocardial death initiates at the endocardium and extends towards the epicardium over time. Hence, strategies to facilitate faster reperfusion are paramount in saving myocardial function, leading to the adage among clinicians that “time is muscle.”

However, the benefit of early reperfusion was challenged as far back as the 1950–1960s by reports of a paradoxical increase in tissue injury following reperfusion in various animal models and was subsequently recognized as IRI. The premise of the molecular mechanisms responsible for IRI is complex and involves multiple cellular components. The initial hypoxia generated by a loss of blood flow causes a decrease in oxidative phosphorylation, a decrease in cellular ATP, and a loss of mitochondrial membrane depolarization. Additionally, the change to anaerobic glycolysis within the myocardium causes a decrease in intracellular pH due to the accumulation of lactate, an end-product of anaerobic respiration. The intracellular accumulation of protons activates the Na^+^/H^+^ exchanger, which expels protons at the expense of accumulating Na^+^. This increase in intracellular Na^+^ coupled with the cessation of mitochondrial ATP synthesis results in the inhibition of the Na^+^/K^+^ ATPase and the subsequent reversal of Na^+^/Ca^2+^ exchanger function, leading to cytosolic Ca^2+^ overload^31^.

While reperfusion restores oxygen delivery, thereby stimulating oxidative phosphorylation and ATP production, re-establishing respiratory chain function facilitates mitochondrial Ca^2+^ influx. Moreover, the sudden reintroduction of oxygen-rich blood to a hypoxic tissue depleted of oxygen scavengers triggers an unregulated ROS-mediated cascade, leading to accelerated tissue necrosis, which can continue for up to 3 days after the onset of reperfusion.

Mitochondrial dysfunction caused by IRI contributes significantly to myocardial damage^5^. More specifically, the conditions of high cytosolic Ca^2+^ leading to mitochondrial Ca^2+^ overload, combined with unregulated ROS production, trigger the opening of the MPTP, a nonselective inner membrane channel. MPTP opening causes the permeabilization of the mitochondrial inner membrane, leading to mitochondrial depolarization, swelling, rupture, and cell death^32–35^. Hence, the MPTP has been a therapeutic target of interest in preventing IRI. Animal studies^36–38^ as well as small clinical trials have demonstrated that cardiomyocyte death and myocardial infarct size can be significantly reduced through pharmacological agents that prevent opening of the MPTP during reperfusion. Cyclophilin D is one such regulatory component of the MPTP and has been targeted through pharmacological agents such as cyclosporine A. While preclinical and early-phase trials of cyclosporine A have shown promise, a larger phase III clinical trial (the CIRUS clinical trial of patients with acute anterior ST-segment elevation myocardial infarction given cyclosporine before undergoing percutaneous coronary intervention) has not been as successful^39,40^. Given that direct inhibition of the MPTP may pose challenges, targeting MPTP activators such as ROS may be a therapeutic strategy of interest.

### Heart failure

While IRI-related ROS injury is acute and occurs in a matter of hours to days, ROS-related effects in HF are more chronic. ROS production in HF has been attributed to chronic neurohormonal activation and consequent upregulation of angiotensin II^41^ as well as increased myocardial stresses associated with pressure overload or hypoxia^42^. HF produces a complex cardiac phenotype involving a combination of cardiomyocyte hypertrophy, fibrosis, arrhythmia, and contractile dysfunction^43^. NOXs have been implicated in many of these phenotypes, and NOX expression is upregulated in numerous independent studies of HF^44,45^. Within the NOX family, NOX2 and 4 are most abundant within the cardiomyocyte. Angiotensin II has been shown to upregulate ROS production in cardiomyocytes through NOX2, causing cardiomyocyte hypertrophy, and profibrotic and proinflammatory changes that lead to remodeling within the cardiovascular system^46^. While NOX2 causes cardiac hypertrophy through angiotensin II, NOX4, which partially localizes to the mitochondria, is associated with cardiac hypertrophy due to increased pressure overload from myocardial stresses in the failing human heart. While NOX4 is found to increase myocardial angiogenesis and protect against contractile dysfunction in HF^42^, recent evidence suggests that NOX4 in failing heart models undergoes alternative splicing, which may explain its detrimental involvement in HF^47^. Other studies have shown that NOX4 depletion decreased mitochondrial swelling and cytochrome *c* release and decreased mitochondrial DNA (mtDNA) damage. These contradictory results leave many uncertainties related to the role of NOX4 in HF.

The role of NOXs in HF continues to draw widespread interest due to their potential as mitochondrial-specific therapeutic targets for disease modulation.

#### Diabetic cardiomyopathy

Cardiomyopathy in diabetic patients is associated with metabolic abnormalities related to high levels of circulating fatty acids as well as elevated fatty acid stores within cardiomyocytes. Up to 70% of the ATP generated by the healthy heart is produced through the oxidation of fatty acids^48^, while the remaining energy is generated by carbohydrates. Healthy cardiomyocytes use their energy sources flexibly to match ATP demands and oxygen availability. Fatty acid β oxidation (FAO), which mainly takes place in the mitochondria and peroxisomes, requires high oxygen availability and is inefficient in times of high ATP needs. In diabetic hearts, due to high circulating and stored fatty acids, FAO continues to be a predominant source of ATP, and there is a relative loss in energy contribution from carbohydrates and glucose during periods of high ATP demand^48^. The loss in flexibility between energy sources causes reduced cardiac efficiency and contractile dysfunction, which is a hallmark of diabetic cardiomyopathy. Peroxisome proliferator-activated receptors (PPARs) are nuclear hormone receptors that largely modulate glucose and fatty acid metabolism and have been implicated in diabetic cardiomyopathy. Animal studies suggest that elevated levels of PPAR-γ and decreased levels of PPAR-α in diabetic cardiomyopathy alter glucose transportation and increase fatty acid accumulation within the cardiomyocyte, thereby altering FAO kinetics^49^. Furthermore, PPAR contributes to a decrease in mitochondrial oxidative phosphorylation and causes downstream effects that regulate mitochondrial ultrastructure and number. An increase in FAO causes increases in mitochondrial inner membrane potential and stimulates ROS^50,51^, which further causes oxidative damage of mitochondrial proteins and DNA, making them less efficient in energy metabolism. Mitochondria in diabetic cardiomyopathy are further compromised due to chronic upregulation of uncoupling proteins found within the inner mitochondrial membrane that adversely affect mitochondrial energetics and calcium stores^52^. The cumulative damage to mitochondria caused by metabolic derangements in diabetes further upregulates ROS production that promotes apoptosis, as suggested by animal studies^53^. Over time, apoptotic cardiomyocytes are replaced by fibroblasts, and this change in tissue composition has been implicated in HF with preserved ejection fraction^54^.

## A specific role for mitochondrial ROS in cardiac disease pathogenesis: lessons from animal modeling

The central role of mitochondrial ROS and heart disease is highlighted by a number of genetic models in which the modulation of either mitochondrial ROS production pathways or mitochondrial ROS scavenging systems has a significant impact on cardiac physiology and the development of cardiac disease (summarized in Table 1). One striking example that demonstrates the interconnectivity between mitochondrial respiratory chain dysfunction, mitochondrial oxidative stress, and cardiac dysfunction is the mitochondrial polymerase γ mutant mice (the “mutator” model). DNA polymerase γ is a mitochondrial polymerase responsible for mtDNA replication. Mutator mice harbor a homozygous D257A substitution in the catalytic PolgA subunit that renders the polymerase proofreading deficient, leading to the progressive accumulation of mtDNA mutations and deletions and premature aging^55,56^. Of note, mutator animals develop accelerated age-associated cardiomyopathy, characterized by increased hypertrophy, ventricular dilation, fibrosis, and cardiac dysfunction^56^. While this accelerated aging observed in the mutator animals has been suggested to be due in part to mitochondrial dysfunction induced by the accumulation of mtDNA lesions^57^, mitochondrial oxidative damage has also been suggested to play a role^56,58^. The contribution of mitochondrial ROS to the cardiac aging phenotype is underscored by the observations that the treatment of mutator mice with SkQ1 (10-(6′-plastoquinonyl)decyltri-phenylphosphonium cation, a mitochondrial antioxidant) improves cardiac mitochondrial ultrastructure^58^ and that enhancing mitochondrial ROS scavenging with a mitochondria-targeted catalase ameliorates the exaggerated aging-associated cardiomyopathy^56^.

**Table 1:** Summary of animal models of mitochondrial ROS and cardiac disease pathogenesis.

Genes	Animal	Phenotype	References
Mitochondrial polymerase γ	Mouse (mutator model)	mtDNA mutations and deletions	Trifunovic et al.^55^
		Premature aging	Dai et al.^56^
		Accelerated age-associated cardiomyopathy (hypertrophy, ventricular dilation, fibrosis, and cardiac dysfunction)	Kolesar, 2014
p66shc deletion	Mouse DC	Ameliorates diabetes-induced cardiac remodeling and normalizes cardiac function	Rota et al.^61^
MAO-A deletion	Mouse TAC	Reduced ventricular dilation and interstitial fibrosis Preserved cardiac function	Kaludercic et al.^65^
	Mouse IRI	Cardioprotective	Pchejetski,2007 Bianchi et al.^67^
MOA-B deletion	Mouse TAC	Robust concentric hypertrophy but inhibits the transition to heart failure over time	Kaludercic et al.^66^
Nox4 deletion	Mouse TAC	Conflicting results	Kuroda et al.^20^ Zhang et al.^42^
MnSOD homozygous knockout	Mouse	Neonatally lethal Left ventricular dilation, cardiomyocyte hypertrophy, and fibrosis	Li et al.^7^
MnSOD heterozygous knockout	Mouse	Phenotypically normal at baseline Cardiac mitochondria sensitized to MPTP activation and ROS-induced cardiomyocyte death	Van Remmen et al.^71^
MnSOD overexpression	OVE26 mouse DC	Protective effect	Shen et al.^72^
Gpx1 loss	Mouse CH	Increased cardiac hypertrophy	Ardanaz et al.^8^
	Mouse IRI	Increased infarct sizes	Chen et al.^9^
Gpx1 overexpression	Mouse MI	Enhanced survival Infarct size was not reduced Enhanced ventricular function Less cardiomyocyte death	Shiomi et al.^74^
Trx2 depletion	Rat	Cardiomyocyte hypertrophy	Hu et al.^10^
Trx2 deletion	Mouse	Dilated cardiomyopathy Severe decline in cardiac contractility Early mortality	Huang et al.^11^
Prx3 overexpression	Mouse MI	Reduced hypertrophy, fibrosis and cardiomyocyte death Increased survival	Matsushima et al.^75^
Mitochondrial catalase overexpression	Mouse CH	Reduced cardiac hypertrophy and fibrosis Improved cardiac function	Dai et al.^76^

*DC* diabetic cardiomyopathy, *IRI* ischemia–reperfusion injury, *MI* myocardial infarction, *CH* cardiac hypertrophy, *TAC* transaortic constriction

In addition to respiratory chain dysfunction-mediated ROS dysregulation leading to cardiac disease, genetic models targeting p66shc, MAO, and NOX4 also support a specific role for mitochondrial ROS in cardiac disease. As mentioned above, mitochondrially localized p66shc mediates the production of hydrogen peroxide and participates in oxidative stress signaling. As elevated mitochondrial ROS can trigger the activation of apoptosis and MPTP-dependent death, the mitochondrial localization of p66shc has been proposed to play an important role in the cellular response to death stimuli. Indeed, cells lacking p66shc display reduced ROS^59^ and are protected against a wide variety of proapoptotic stimuli, including hydrogen peroxide, ultraviolet irradiation, and staurosporine^18,60^. This protection conferred by p66shc deletion in vitro has translated into some protective effects in the heart. p66shc-knockout animals are protected in the streptozotocin model of diabetic cardiomyopathy. The loss of p66shc ameliorates diabetes-induced cardiac remodeling and normalizes cardiac function^61^. p66shc has also been implicated in the regulation of cardiomyocyte loss following cardiac IRI, although in this setting the results have been less defined. While ex vivo IR studies have demonstrated cardioprotection with p66shc deletion^62^, in vivo IR studies have yielded the opposite effect^63,64^. Together, these studies suggest that the impact of p66shc on cardiac pathology may be disease-dependent.

Similar to p66shc, both isoforms of MAO, MAO-A, and MAO-B, are sources of mitochondrial hydrogen peroxide. In vivo studies have demonstrated clear contributions of MAO-A and MAO-B to HF and cardiac IRI. In the mouse transverse aortic constriction (TAC) model of chronic pressure overload-induced cardiac hypertrophy and HF, inhibition of MAO-A with clorgyline (a MAO-A-specific inhibitor) reduces oxidative stress, hypertrophy, and cardiomyocyte death^65^. Similar to the effects observed with clorgyline, genetic inactivation of MAO-A is also protective, as mice lacking MAO-A display reduced ventricular dilation and interstitial fibrosis with preserved cardiac function in response to TAC^65^. Similar to MAO-A, the loss of MAO-B is also protective against cardiac pressure overload, but the role of MAO-B may be more nuanced. In response to TAC, MAO-B-deficient animals develop robust concentric hypertrophy, but over time, the transition to fulminant HF is inhibited^66^. Collectively, these studies suggest that MAO-A and MAO-B might control different aspects of pressure overload-induced cardiac remodeling and failure. Both the deletion and pharmacological inhibition of MAO-A have also been demonstrated to be cardioprotective in cardiac IRI^67^. Most importantly, the protection conferred by MAO-A inactivation is present even when MAO inhibitors are administered after the onset of ischemia^67^, suggesting that MAO inhibitors might be ideal for use in a clinical setting.

As mentioned above, NOX4 partially localizes to the mitochondria and can be a source of mitochondrial superoxide. NOX4 has come into focus in the setting of cardiac disease, as NOX4 expression has been found to be elevated in HF, atrial fibrillation, and atherosclerosis^68–70^. Cardiac-specific NOX4-knockout mouse models have demonstrated that mitochondrial NOX4 is integral to mitochondrial dysfunction, cardiomyocyte apoptosis, and left ventricular dysfunction through increased production of superoxide, leading to oxidative modification of mitochondrial proteins, which contributes to cardiac dysfunction^20^. While there have been conflicting reports of the impact of NOX4 in mouse models of cardiac pressure overload^20,42^, the coincident upregulation of NOX4 with cardiac disease is highly suggestive that NOX4-mediated ROS production contributes to disease pathogenesis.

The dysregulation of mitochondrial ROS scavenging systems also provides strong support for a key role of mitochondrial ROS in cardiac disease. The pathological consequences of impaired MnSOD-mediated ROS detoxification are clear examples. MnSOD homozygous knockout mice are neonatal lethal, dying by postnatal day 10 with dilated cardiomyopathy characterized by left ventricular dilation, cardiomyocyte hypertrophy, and fibrosis^7^. While mice heterozygous for the loss of MnSOD are phenotypically normal at baseline, cardiac mitochondria from these animals are sensitized to MPTP activation and ROS-induced cardiomyocyte death^7,71^. In contrast to the deleterious effects of MnSOD depletion, cardiomyocyte overexpression of MnSOD is protective in the OVE26 mouse model of type 1 diabetes and diabetic cardiomyopathy^72^.

Reduction in GSH-PX activity has also been found to impact cardiac disease progression. Mice lacking Gpx1 display increased cardiac hypertrophy in response to prolonged angiotensin II infusion and display increased infarct sizes following IRI^8,9^, suggesting that the loss of Gpx1 potentiates cardiac disease. However, the effects of Gpx1 ablation on IRI may be more complex, as it has been observed that Gpx1 ablation sensitizes male mice to IRI, but female mice are protected through a pathway that may involve enhanced nitrate consumption and nitric oxide production^73^. Nonetheless, mice overexpressing Gpx1 display enhanced survival following myocardial infarction, and while infarct size was not reduced, Gpx1-overexpressing mice display enhanced ventricular function with less cardiomyocyte death^74^.

Similar to the reduction in GSH-PX activity, the disruption of the PRX/Trx-mediated ROS scavenging through the depletion of Trx2 neonatal rat ventricular cardiomyocytes causes cardiomyocyte hypertrophy^10^, and mice with cardiomyocyte-specific deletion of Trx2 develop dilated cardiomyopathy with a severe decline in cardiac contractility and early mortality^11^. Conversely, enhancing this system through the overexpression of Prx3 resulted in mice with reduced hypertrophy, fibrosis, and cardiomyocyte death with increased survival following myocardial infarction^75^.

Finally, the cardioprotective effects of mitochondria-specific ROS scavenging were directly demonstrated by studies comparing the effects of enforced cytosolic versus mitochondrial expression of catalase. While both cytosolic and mitochondria-targeted catalase effectively reduced oxidative stress, only mice overexpressing mitochondrial catalase (but not mice overexpressing cytosolic catalase) displayed significantly reduced cardiac hypertrophy and fibrosis with improved cardiac function in the angiotensin II model of cardiac hypertrophy^76^. These protective effects of mitochondrial catalase expression extended to the TAC model of cardiac hypertrophy and HF. Importantly, alterations of the mitochondrial proteome due to chronic pressure overload are attenuated with mitochondrial catalase-mediated ROS detoxification^77^. Together, these studies provide strong support for a central role for mitochondrial ROS in potentiating cardiac disease and for mitochondrial ROS scavenging as an avenue for therapy.

## ROS as a therapeutic target for heart disease

In light of the strong links between elevated ROS, oxidative stress, and cardiac disease, ROS has emerged as an attractive target for therapy, with the goal of many drug-based strategies being to boost cellular antioxidant capacity and enhance ROS detoxification (summarized in Table 2). Here, we highlight some of the major clinical trials evaluating the impact of general antioxidant strategies on heart disease. A number of smaller studies have shown some positive effects of global antioxidants, such as vitamin C, vitamin E, and *N*-acetylcysteine (NAC), on the treatment of heart disease. However, studies examining the impact of vitamin C and vitamin E with expanded patient cohorts do not support any beneficial effects of antioxidant vitamin supplementation, and the confirmation of the effects of NAC on larger patient cohorts is still outstanding (Table 1).

**Table 2:** Summary of selected antioxidant therapies for cardiovascular diseases.

Therapy	Disease	Model	Summary	References
Global antioxidants				
Vitamin C	CVD CHF	Human	Ineffective or harmful	Cook et al.^82^, Nightingale et al.^86^
Vitamin E	CVD CHD	Human	Ineffective	Lee et al.^83^, Tornwall et al.^85^, Virtamo et al.^84^
NAC	MI	Human	Reduced infarct size and enhanced ventricular function	Sochman et al.^87^, Arstall et al.^89^, Yesilbursa et al.^90^, Pasupathy et al.^91^
Mitochondria-targeted antioxidants				
Therapy	Disease	Model	Summary	References
MitoTEMPO	CHF	Guinea pig	Decreased mitochondrial and cytosolic ROS	Dey et al.^93^
	Chronic pressure overload; DC	Mouse	Improved mitochondrial respiration, reduced hypertrophy, and improved cardiac function	Hoshino et al.^94^
MitoQ	IRI	Rat	Decreased mitochondrial damage and cardiomyocyte death Preserved mitochondrial membrane potential and respiration	Adlam et al.^98^ Ribeiro Junior et al.^99^
SS-31/MPT-131/Bendavia/ Elamipretide	IRI	Rabbit	Reduced infarct size and microvascular damage	
	CHF	Mouse	Reduced infarct size and fibrosis; re-established mitochondrial ultrastructure and proteome	Kloner et al.^105^, Cho et al.^106^, Brown et al.^107^, Dai et al.^108^
	IRI	Dog	Reduced infarct size; decreased ROS; increased ejection fraction; preserved mitochondrial function	Sabbah et al.^109^
	IRI	Rat	Reduced infarct size	

*CHD* coronary heart disease, *CHF* chronic heart failure, *CVD* cardiovascular diseases, *DC* diabetic cardiomyopathy, NAC *N*-acetylcysteine, *IRI* ischemia–reperfusion injury, *MI* myocardial infarction

### Ascorbic acid (vitamin C) and α-tocopherol (vitamin E)

Vitamin E is a lipid-soluble antioxidant that can terminate lipid peroxidation chain reactions by stabilizing lipid peroxyl radicals, while vitamin C is a water-soluble radical-scavenging antioxidant with the ability to regenerate vitamin E in cellular membranes. Clinical trials using these antioxidant vitamins to decrease oxidative stress in cardiovascular disease have yielded inconsistent results. Initial studies, with numbers of participants ranging from 10 to 55, evaluating the effect of vitamin C in chronic HF showed promising results^78,79^. Early studies assessing the impact of vitamin E treatment on coronary disease risk in men and women suggested that higher vitamin E intake lowers the risk of coronary disease^80,81^. However, more recent studies with larger patient cohorts have not confirmed earlier findings. In a randomized trial analyzing the impact of vitamin C, vitamin E, and β-carotene in 8171 women at high risk for cardiovascular disease (either with a history of cardiovascular disease or presenting with at least three cardiovascular disease risk factors), treatment with these supplements had no effect on preventing the future occurrence of cardiovascular events^82^. Moreover, a host of additional studies also conclude that vitamin C and/or vitamin E, given intravenously or orally, has little to no effect on the occurrence of cardiovascular events in healthy women^83^ and male smokers^84,85^. Moreover, instead of being protective, vitamin C treatment may worsen muscle metabolism in chronic HF patients^86^. Finally, a meta-analysis of 15 independent randomized controlled trials encompassing 188,209 patients assessing the effect of vitamin antioxidant supplementation and the prevention of cardiovascular events revealed that vitamin administration had no effect on the incidence of cardiovascular events^12^. Together, these results indicate that the global antioxidants vitamin C and E have little to no effect on heart disease.

### *N*-acetylcysteine

NAC is a thiol-containing antioxidant that has the ability to regenerate intracellular antioxidant pools, as NAC deacetylation produces cysteine, which is a precursor for reduced GSH. Initial small trials evaluating the efficacy of intravenous NAC on acute MI and ischemic heart disease have shown promise. In the 30 patient ISLAND (Infarct Size Limitation: Acute NAC Defense) trial to assess the effect of NAC on reperfusion injury, NAC administration in combination with streptokinase-mediated recanalization reduced infarct size and improved left ventricular EF^87^. In addition to ROS scavenging, NAC has the ability to potentiate the vasodilation, hemodynamic, and anticoagulation effects of nitroglycerin;^88^ thus, the combinatorial use of NAC together with nitroglycerin has been a strategy of great interest. Preliminary trials assessing the effect of NAC together with nitroglycerin and streptokinase have been promising, with patients given NAC displaying reduced oxidative stress and enhanced ventricular function^89,90^. Moreover, the subsequent NACIAM (NAC in Acute Myocardial Infarction) trial, evaluating the use of high-dose NAC in combination with low-dose nitroglycerin in ST-segment elevation myocardial infarction patients with percutaneous coronary intervention, also showed that combined NAC and nitroglycerin treatment could significantly reduce infarct size and increase myocardial salvage^91^. While the success of these preliminary clinical trials with limited number of patients is encouraging, larger multicenter clinical trials are needed to determine if the beneficial effects of combined NAC and nitroglycerin treatment can be replicated and broadly applied.

## Mitochondria-targeted antioxidant therapies

In light of the limited success of general antioxidants in mitigating heart disease and the strong preclinical animal studies supporting the idea that limiting ROS production or enhancing ROS scavenging in the mitochondrial compartment can be highly beneficial to the heart, in recent years, considerable effort has been directed towards developing mitochondria-targeted antioxidants as pharmacological agents to ameliorate disease. MitoTEMPO, mitoQ, and SS-31/MPT-131 are some of the mitochondria-targeted agents that have garnered the most interest.

### MitoTEMPO

MitoTEMPO is a mitochondria-targeted SOD mimetic that consists of piperidine nitroxide (2,2,6,6-tetramethylpiperidine-*1*-oxy) conjugated to the lipophilic triphenylphosphonium cation (TPP^+^) that facilitates the membrane potential-dependent accumulation of the compound into the matrix^92^. MitoTEMPO detoxifies superoxide by cycling between its nitroxide and oxoammonium forms, as well as oxidizing ferrous iron to limit hydroxyl radical formation^92^. MitoTEMPO has been studied in a wide range of animal models of cardiac disease. In the guinea pig ascending aortic constriction/β-adrenergic stimulation model of HF and sudden cardiac death, mitoTEMPO effectively reduced ROS production in both mitochondrial and cytosolic compartments in failing myocytes, and chronic administration prevented HF^93^. Importantly, the administration of mitoTEMPO after the onset of cardiac hypertrophy was protective, suggesting that cardiac remodeling can be reversed and that mitochondrial ROS scavenging can be deployed as a therapy following the onset of disease^93^. The protective effects of mitoTEMPO extend to the murine TAC model of chronic pressure overload as long-term mitoTEMPO administration markedly improves both mitochondrial respiratory chain function and cardiac contractile performance^94^. Finally, mitoTEMPO has also been shown to be beneficial in the setting of diabetic cardiomyopathy, as it has been demonstrated to reduce hypertrophy and preserve cardiac function in the streptozotocin and db/db mouse models of type 1 and type 2 diabetes^95^.

### Mitoquinone

MitoQ (mitoquinone) is a mitochondrial matrix-targeted ubiquinone appended to the TPP^+^ cation. Once targeted to the matrix, ubiquinone is reduced to ubiquinol, which functions as both an electron carrier to facilitate electron transfer between complexes I/II to complex III and as an antioxidant by decreasing lipid peroxidation^96^. In vitro, mitoQ effectively reduces oxidative damage and protects against cell death induced by hydrogen peroxide as well as chemically induced IR^96,97^. In vivo, rats administered mitoQ in drinking water display reduced mitochondrial damage, cytochrome *c* release, caspase 3 activation, and cardiomyocyte death in a Langendorff isolated heart model of IRI^98^. Importantly, this reduction in cardiomyocyte loss was accompanied by enhanced cardiac contractility and preserved mitochondrial respiratory chain function, suggesting that mitoQ is both mitoprotective and cardioprotective^98^. In line with its mitoprotective nature, mitoQ-treated rats subjected to prolonged TAC also displayed preserved mitochondrial membrane potential and respiratory chain function as well as reduced sensitivity to oxidant-induced MPTP^99^. However, while mitoQ reduced right ventricular hypertrophy and lung edema, mitoQ treatment had no effect on cardiac function^99^. Taken together, these studies suggest that mitoQ-mediated ROS scavenging may be most efficacious when used in specific disease contexts, such as cardiac IRI.

### SS-31/MPT-131/Bendavia/Elamipretide

Unlike mitoTEMPO or mitoQ, which require the TPP^+^ cation and mitochondrial membrane potential for mitochondrial targeting, SS-31 (d-Arg-2′6′-dimethylTyr-Lys-Phe-NH2) and its acetate salt MTP-131 (also known as Bendavia or Elamipretide) are mitochondria-targeted antioxidant peptides that harbor alternating aromatic-cationic motifs that facilitate high solubility and membrane permeability^100^. SS-31/MPT-131 is unique in its ability to accumulate at the mitochondrial inner membrane in a membrane potential-independent fashion, thus allowing the possibility of this compound to act on both functional and diseased mitochondria. At the inner membrane, SS-31 binds to cardiolipin, which is an inner membrane-specific phospholipid with a central role in the regulation of cristae architecture, mtDNA nucleoid distribution, respiratory chain complex integrity, and supercomplex organization^101^. SS-31 has been shown to specifically modulate cardiolipin’s interaction with cytochrome *c*^102^. While the main function of cytochrome *c* in the respiratory chain is to shuttle electrons between complex III and complex IV, cytochrome *c* can also function as a peroxidase and thereby mediate mitochondrial oxidative damage. This balance between the electron carrier and peroxidase activities of cytochrome *c* is controlled by cardiolipin. Hydrophobic binding between cardiolipin and cytochrome *c* facilitates a partial unfolding of cytochrome *c* that promotes peroxidase activity. SS-31 works by binding to cardiolipin and disrupting the cardiolipin–cytochrome *c* interaction, thereby promoting the function of cytochrome *c* as a respiratory chain electron carrier^103^.

SS-31 has been widely studied, and in vitro, it has been found to limit mitochondrial ROS production, reduce oxidative damage, and improve mitochondrial bioenergetics^104^. In vivo, SS-31 has shown incredible promise to limit myocardial damage and promote cardiac function in animal models of ischemic injury and HF. In the context of cardiac IRI, the administration of SS-31 either at the onset of ischemia or even just prior to reperfusion significantly reduces infarct size in mouse, rat, guinea pig, and rabbit models^105–107^. Additionally, SS-31 has been demonstrated to reduce the extent of microvascular damage in an ex vivo rabbit model of IR, collectively supporting the concept that mitochondrial ROS plays a significant role in reoxygenation-induced myocardial damage and that SS-31 is cardioprotective. In the setting of HF, SS-31 restricts cardiac hypertrophy, reduces fibrosis, improves cardiac function in mice subjected to TAC^108^, and, importantly, profoundly reduces the degree of mitochondrial ultrastructural and proteomic changes that occur^108^. Similar to the cardioprotective effects observed in mice, Elamipretide was highly protective in a canine model of microembolism-induced HF. Dogs subjected to a 3-month chronic daily Elamipretide regimen following HF onset displayed decreased ROS burden, enhanced cardiac EF, a normalization of serum biomarkers of HF, as well as preserved mitochondrial function^109^. Collectively, these animal studies suggest that mitochondrial oxidative damage is a fundamental pathogenic mechanism in both IRI and HF and that targeting mitochondrial oxidative stress through cardiolipin would be of great therapeutic benefit.

## Moving from the bench towards the bedside: mitochondria-targeted antioxidants in the clinical setting

In light of the promising results of drug-based mitochondrial ROS scavenging in animal models of heart disease, there has been intense interest in translating these benefits into patients. SS-31 has garnered the most interest due to its efficacy in numerous preclinical animal trials. However, the translation of the benefits of SS-31 into use in human patients has proven challenging. Recently, Elamipretide was tested in a double-blind and placebo-controlled trial in patients presenting with HF with reduced EF^13^. In this study, patients were treated with a single intravenous infusion of Elamipretide, and cardiac function was evaluated for 24 h following drug administration^13^. Importantly, patients treated with high-dose Elamipretide displayed a significant decrease in left ventricular end diastolic and end systolic volumes^13^, suggesting that Elamipretide can improve cardiac function in the context of HF. However, in the EMBRACE STEMI multicenter phase IIa trial to assess the effect of MPT-131 in patients with ST-elevated myocardial infarction, MPT-131, while safe and well tolerated in patients, had no significant impact on infarct size or ventricular function^110^. Taken together, the safety profile of SS-31-based compounds in patients, together with the preliminary success with acute treatment in the setting of HF with reduced EF, suggest that antioxidant peptide-mediated mitochondrial ROS scavenging holds promise for use in the clinical setting. However, the EMBRACE STEMI trial highlights the challenges of translating drug efficacy in animal studies to benefits in patients. Elucidating the optimal dosing, timing, and duration of drug administration and understanding whether these parameters are specific for different types of cardiac disease will need to be at the forefront of research efforts as we move to bring mitochondria-targeted antioxidant therapy from the bench to the bedside.

## Discussion

In recent decades, significant progress has been made in developing therapeutics to preserve mitochondrial integrity and attenuate oxidative stress in heart disease. Many of these potential treatments show great promise in vitro, and one of the greatest challenges we now face is translating efficacy in preclinical animal studies into therapies for ROS-mediated cardiomyopathies in human patients. In making the transition from the bench to the bedside, our understanding of some key issues will need to be expanded. Critically, we will need to understand what types of heart disease would most benefit from mitochondrial antioxidant therapy and if there are some disease modalities that would benefit from global versus mitochondria-targeted strategies. In addition, in the design of therapeutic regimens, it will be essential to know at what stages of disease development mitochondrial antioxidant therapies should be optimally deployed. Moreover, as the mitochondrial ROS pool plays a significant role in disease pathogenesis, the development of compounds with improved mitochondrial targeting and enhanced antioxidant activity will be key to oxidative stress-based approaches to treating human cardiovascular diseases. In addition to new and more efficacious mitochondrial ROS scavenging compounds, given that ROS can play a role in both physiological and pathological signaling, understanding the differences between these two modes and developing strategies to mitigate pathological ROS damage while preserving physiological ROS signaling will be an important next challenge.
